# Molecular epidemiology of vancomycin resistant enterococci collected from 9 hospitals in Beijing, China: a multicenter study

**DOI:** 10.3389/fcimb.2026.1730782

**Published:** 2026-06-04

**Authors:** Lanqing Cui, Yun Li, Lingyun Ma, Huimin Ma, Jingxian Yang, Yanan Tan, Chunyue Ge, Dong Li, Yi Wang, Xinying Yang, Xiaoyi Liu, Ronghua Geng, Shumei Liu, Xiangyan Li, Bo Zheng

**Affiliations:** 1 Institute of Clinical Pharmacology, Peking University First Hospital, Beijing, China; 2 Department of Infectious Disease, Peking University First Hospital, Beijing, China; 3 Department of Clinical Laboratory, Aerospace Center Hospital, Beijing, China; 4 Department of Clinical Laboratory, Beijing Haidian Hospital, Beijing, China; 5 Department of Clinical Laboratory, Beijing Hospital, National Center for Gerontology; National Clinical Research Center for Gerontology; The Key Laboratory of Geriatrics of NHC; Institute of Geriatric Medicine, Chinese Academy of Medical Sciences, Beijing, China; 6 Department of Clinical Laboratory, Civil Aviation General Hospital, Beijing, China; 7 Department of Clinical Laboratory, Beijing Bo’ai Hospital, China Rehabilitation Research Center, Beijing, China; 8 Department of Clinical Laboratory, Beijing No.6 Hospital, Beijing, China; 9 Department of Clinical Laboratory, The First Hospital of Tsinghua University, School of Clinical Medicine, Tsinghua Medicine, Tsinghua University, Beijing, China; 10 Department of Clinical Laboratory, Aviation General Hospital, Beijing, China; 11 Department of Clinical Laboratory, Fuxing Hospital, Capital Medical University, Beijing, China

**Keywords:** molecular epidemiology, ST80, vanA, vancomycin resistant enterococci, vanM

## Abstract

**Objective:**

To investigate the antibacterial susceptibility and molecular characteristics of vancomycin resistant enterococci (VRE) isolated from 9 hospitals in Beijing from November 2021 to October 2022.

**Methods:**

The susceptibilities to 17 antibacteria agents were tested using two-fold agar dilution and broth microdilution. The resistance genes were characterized through whole genome sequence analysis. The bacteria typing was investigated through multilocus sequence typing (MLST), core genome MLST(cgMLST).

**Results:**

A total of 251 VRE isolates including 247 *Enterococcus faecium* (VREfm) and 4 *Enterococcus faecalis* (VREfs) were collected in this study. More than 75% isolates were collected from urine. More than 89% isolates were resistant to teicoplanin. Besides glycopeptides, all the VRE isolates were resistant to penicillin, ampicillin and fluoroquinolones (VREfm) or erythromycin and fluoroquinolones (VREfs) and can be defined as multi-drug resistant. Among 247 VREfm, 72.9% (180/247) carried *vanA* gene, 0.8% (2/247) carried *vanM* gene, and 26.3% (65/247) carried both *vanA* and *vanM* genes. A total of 22 sequence types (STs) were identified, with ST80 (24.7%, 61/247) being the most predominant. cgMLST analysis revealed that ST80-CT8524 was the most common cluster among the VREfm isolates. All the 4 VREfs isolates harbored *vanA* gene and were assigned to 2 STs.

**Conclusion:**

VREfm isolates belonging to ST80 in Beijing was most common. *VanA* was still the predominant *van* gene and increased coexistence of *vanA* and *vanM* was noteworthy.

## Introduction

Enterococci were important healthcare-associated pathogen and can cause many infections including urinary tract infections, skin and soft issue infections, bloodstream infections (Miller et al., 2020). Vancomycin, the typical glycopeptide antibiotic, remains as an important choice for the treatment of serious infections caused by resistant enterococci. However, vancomycin resistant *Enterococci* (VRE) has been found worldwide since first identified in Europe in 1980s and now has become a major threat to the public health due to lack of effective treatment and high mortality (Werner et al., 2020). Compared to *Enterococcus faecalis* (*E. faecalis*), vancomycin resistance among *Enterococcus faecium* (*E. faecium*) was more serious. World Health Organization has listed vancomycin resistant *Enterococcus faecium* (VREfm) as one of the global priority pathogens in 2017 (Tokano et al., 2024).

VRE positive rates differed between different countries and in Asia it was about 8.1% which was lower than the United states and higher than most European countries (Shrestha et al., 2021). In China, the overall detection rate of VRE was relatively low and was relatively low, remaining below 2% in 2022 according to China antimicrobial resistance surveillance system (CARSS), which covers about 1400 hospitals located in 31 provinces and autonomous regions (http://www.carss.cn/). However, the prevalence of VRE in Beijing were more serious than other regions and showed increased trends year by year and the detection rates of VREfs and VREfm in Beijing were 0.9% and 11.7% according to data from CARSS (http://www.carss.cn/). It is essential to investigate the resistance and molecular epidemiological characteristics of VRE in Beijing for the prevention and control. Previously, some relevant studies were conducted, however, the isolates were collected from one hospital or just from ICU patients (Sun et al., 2019; Yan et al., 2023; Yang et al., 2016). Therefore, we conducted this multi-center study to comprehensively characterize the molecular epidemiology of VRE in Beijing.

## Materials and methods

### Bacteria

This study was organized by Institute of Clinical Pharmacology, Peking University First Hospital involved 9 hospitals in Beijing. The hospitals were listed in descending order of the number of isolates: Aerospace Center Hospital (Hospital G, n=83), Beijing Haidian Hospital (Hospital E, n=46), Beijing Hospital (Hospital L, n=26), Civil Aviation General Hospital (Hospital J, n=25), Beijing Bo’ai Hospital (Hospital A, n=20), Beijing No.6 Hospital (Hospital B,n=15), The First Hospital of Tsinghua University (Hospital H, n=14), Aviation General Hospital (Hospital F, n=12) and Fuxing Hospital, Capital Medical University (Hospital D, n=10). VRE isolates were collected from the microbiology laboratories of these hospitals from November 2021 to October 2022 and then submitted to the central laboratory for further study. Basic patient information (e.g., age, sex, ward type, specimen source) were obtained from medical records. Duplicate samples from the same patient were excluded. The species were initially identified by the participating hospitals using Vitek 2 or MALDI-TOF and confirmed in the central laboratory by 16S rRNA gene sequencing. The strains were stored in -80 °C freezer in the central laboratory.

### Antimicrobial susceptibility testing and analysis

The minimum inhibitory concentrations (MICs) of 15 antibacterial agents (vancomycin, teicoplanin, penicillin, ampicillin, erythromycin, tetracycline, minocycline, ciprofloxacin, levofloxacin, moxifloxacin, chloramphenicol, fosfomycin, nitrofurantoin, rifampin, linezolid) were determined by two-fold agar dilution (Sempere et al., 2022), and MICs of two agents (daptomycin and tigecycline) were determined using broth microdilution. The susceptibility testing results were mainly interpreted according to CLSI M100-S35 guideline, and EUCAST v15.0 criteria was used for tigecycline (CLSI, 2025; EUCAST, 2025). *E. faecalis* ATCC 29212 was used as the quality control strain and all MIC values were within CLSI acceptable ranges.

### Whole-genome sequencing and analysis

Genomic DNA of all the isolates were extracted by the SDS method and then were sequenced on Illumina NovaSeq 6000 platform in Beijing Sinobiocore Biological Technology Co., Ltd. (Beijing, China). Paired-end sequencing with 150 bp read length (PE150) was performed, achieving an average sequencing depth of approximately 150 × coverage per isolate. Raw reads were assembled using Edena v3.131028 and annotation was performed using Prodigal v2.6.

Multilocus sequence typing (MLST) was analyzed using MLST database (https://pubmlst.org/organisms/enterococcus-faecium; accessed on 10 October, 2025) to get the sequence type (ST), then STs were further clustered into clonal complex (CC). Minimum spanning tree of ST data was constructed using BioNumerics software version 7.6 (Applied Maths).

Core genome MLST (cgMLST) analysis of VREfm was performed using SeqSphere+version 10.0.0 (Ridom GmbH, Münster, Germany) based on the scheme including 1423 target genes. Minimum spanning trees were constructed using GrapeTree (v. 1.5.0) with the MSTree V2 algorithm, Isolates with missing alleles ≥10% were excluded. The distance threshold for defining the same cluster was ≤20 alleles differences (de Been et al., 2015). Nodes were colored by sequence type (ST), and STs represented by a single isolate were colored white to reduce visual complexity. Branch lengths are proportional to the number of allelic differences.

Acquired resistance genes in the genome were identified from the Resfinder 4.1 Database (https://cge.food.dtu.dk/services/ResFinder/; accessed on 10 October, 2025).

## Results

### Clinical characteristics

A total of 247 VREfm isolates and 4 VREfs isolates were collected from November 2021 to October 2022. Among VREfm isolates, 77.7% (192/247) and 8.9% (22/247) were isolated from urine and blood, respectively, and other sources included secretion (4.4%, 11/247) and intra-abdominal drainage (3.6%, 9/247). Among the four VREfs isolates, three strains were isolated from urine and one from blood.

### Antibacterial susceptibility and resistance genes

The VREfm isolates included in this study were all resistant to vancomycin with MIC ranging from 64 to >256 ug/mL and 92.3% (228/247) had high vancomycin MIC values (≥256 ug/mL). Of the 247 isolates, 89.1% (220/247) were resistant, 9.3% (23/247) were intermediate, and 1.6% (4/247) were susceptible to teicoplanin (**Table 1**). All VREfm isolates were resistant to penicillin, ampicillin, and fluoroquinolones and were defined as multidrug-resistant (MDR). In addition, more than 70% of isolates were also resistant to erythromycin or tetracycline, and 40.5% of isolates were resistant to tigecycline. Daptomycin and linezolid showed potent activity with resistance rates below 1%. All VREfm isolates carried *van* genes; 72.9% (180/247), 0.8% (2/247), and 26.3% (65/247) carried *vanA* gene, *vanM* gene, both *vanA* and *vanM* gene, respectively. In this study, 55.4% (36/65) of VRE isolates positive for both *vanA* and *vanM* (vanA^+^/vanM^+^) were from hospital E and the percentage of vanA^+^/vanM^+^ isolates in Hospital E was 78.3% (36/46), which varied from 6.3% to 35.7% in other hospitals.

**Table 1:** Susceptibilities of VREfm isolates to antimicrobial agents.

Antibacterial agents	S (%)	I (%)	R (%)	MIC_50_ (mg/L)	MIC_90_ (mg/L)
Penicillin	0.0	0.0	100	>256	>256
Ampicillin	0.0	0.0	100	>256	>256
Vancomycin	0.0	0.4	99.6	>256	>256
Teicoplanin	2.0	9.3	88.7	128	256
Daptomycin	99.6	0.0	0.4	1	2
Erythromycin	7.7	3.6	88.7	>256	>256
Tetracycline	27.9	0.8	71.3	64	256
Minocycline	30.4	8.5	61.1	16	32
Tigecycline	59.5	0.0	40.5	0.12	8
Ciprofloxacin	0.0	0.0	100.0	256	>256
Levofloxacin	0.0	0.0	100.0	128	256
Chloramphenicol	99.6	0.0	0.4	8	8
Fosfomycin	32.8	53.4	13.8	128	256
Nitrofurantoin	15.4	23.1	61.5	128	256
Rifampicin	1.6	1.2	97.2	8	16
Linezolid	98.4	0.8	0.8	2	2

All VREfm isolates carried at least one aminoglycoside resistant gene including *aac(6’)-Ii*, *aac(‘)-*, *(6’)-aph(2’’)*, *aph(3’)-III*, *aph(4’)-III*, *aph(2’’)-Ia*, *aph(2’’)-Ie*, *aph(2’’)-If*, *ant(6)-Ia* and *ant(9)-Ia* and at least one MLS (macrolide, lincosamide and streptogramin B) resistant gene including *msr(C)*, *erm(A)*, *erm(B)*, *erm(T)*, *lsa(A)*, *lsa(E)* and *lnu(B)*. 77.8% (192/247) harbored at least one tetracycline resistance gene including *tet(M)*, *tet(L)* or *tet(S)*. Phenotypically, >70% of isolates were resistant to erythromycin or tetracycline, consistent with the high prevalence of MLS and tetracycline resistance genes described above.

The four VREfs isolates were all resistant to vancomycin and teicoplanin, with MIC ≥ 256mg/L. All were also resistant to erythromycin and fluoroquinolones but susceptible to penicillin, ampicillin, daptomycin, and linezolid. Of these, 50% (2/4) and 25% (1/4) were resistant to tetracycline or tigecycline, respectively. All VREfs carried the *vanA* gene, at least one aminoglycoside resistant gene including *aac(6’)-aph(2’’)*, *aph(3’)-III*, *ant(6)-Ia*, two MLS resistant genes *lsa(A)* and *erm(B)* and one tetracycline resistance gene *tet(M)*.

### MLST analysis

**Figure 1:**
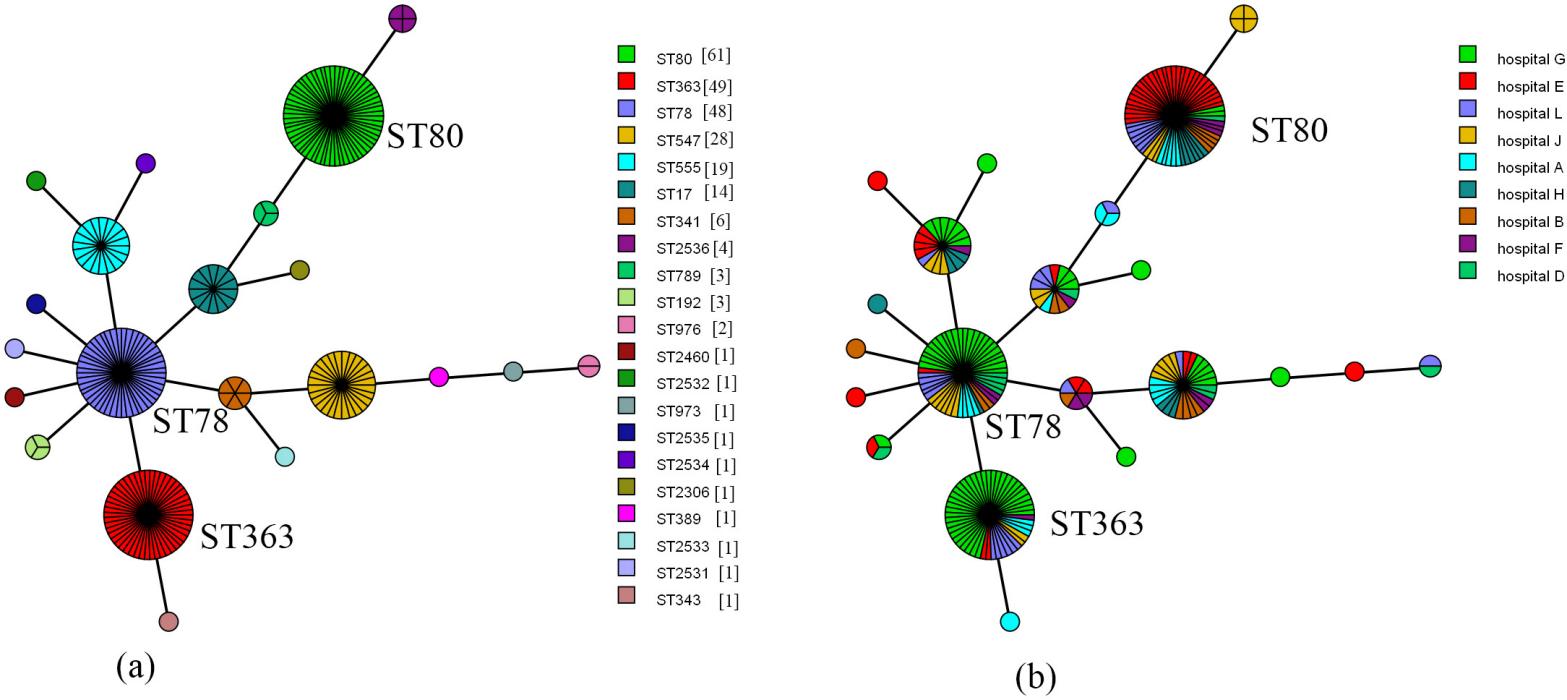
Minimum spanning tree based on MLST data analysis of 247 VREfm isolates. **(a)** Overall structure of 22 STs. Each circle represents a distinct ST, with colors indicating different ST types and circle size proportional to the number of isolates per ST. The number of isolates per ST is indicated in brackets in the legend. The three most prevalent STs are highlighted: ST80 (green), ST363 (red), and ST78 (blue). **(b)** ST distribution across different hospitals, with colors representing individual hospitals. This panel illustrates the presence of STs across hospital settings, revealing that the three predominant STs—ST80, ST78, and ST363—are distributed across multiple hospitals.

MLST analysis revealed that 247 VREfm isolates belonged to 22 STs and 98.7% of isolates belonged to clonal complex (CC) 17. ST80 (24.7%, 61/247), ST363 (19.8%, 49/247), ST78 (19.4%, 48/247), ST547(11.3%, 28/247) and ST555 (7.7%, 19/247) were the five most common types (**Figure 1a**). MLST analysis in different hospitals revealed that among nine participating hospitals, ST80 was the most dominant in six hospitals, except for hospitals D, G and J; the prevalence rates varied from 25.0% in hospital A to 65.2% in hospital E. ST78 was the most dominant in hospital D and G with proportions of 40% and 50%, respectively; while ST363 (43.8%, 35/80) followed by ST78 (27.5%, 22/80) was the most common in hospital J (**Figure 1b**). MLST analysis of strains isolated from urine specimens revealed that ST80 (27.1%, 52/192), ST78 (20.3%, 39/192), and ST363 (18.2%, 35/192) were the three most prevalent sequence types, whereas among strains from blood specimens, the top three STs were ST363 (31.8%, 7/22), ST555 (22.7%, 5/22), and ST78 (18.2%, 4/22), respectively. Six novel STs, including ST2531(15-186-1-1-1-1-1), ST2532 (4-184-1-1-1-1-1), ST2533(4-5-1-1-1-1-1), ST2534 (4-1-1-39-1-1-1), ST2535 (15-185-1-1-1-1-1) and ST2536 (9-1-1-39-12-1-1) were first found in this study and been officially assigned by the PubMLST database.

As for the four VREfs isolates, two isolates belonged to ST6 and the others were assigned ST79 and ST507.

### cgMLST analysis

**Figure 2:**
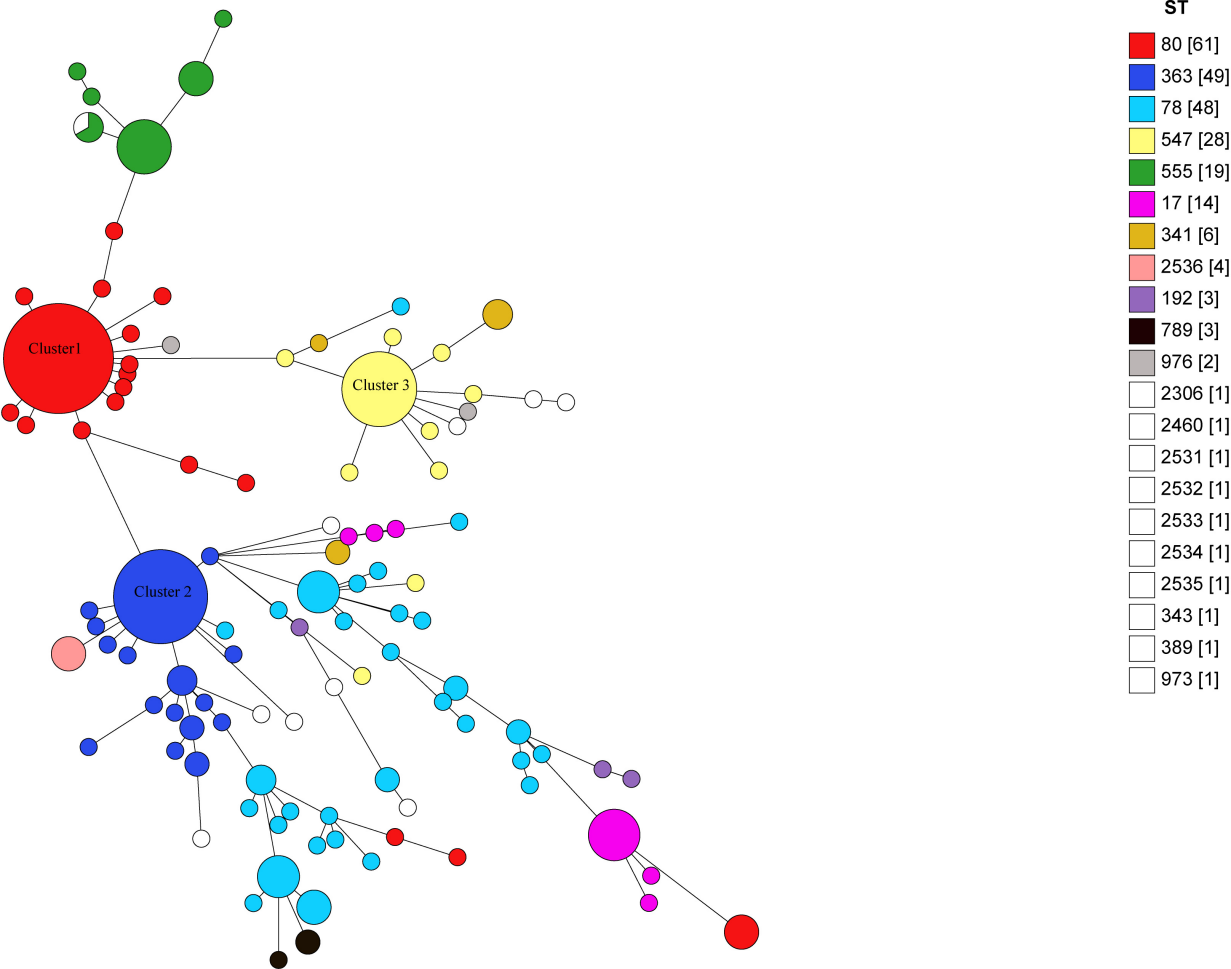
Minimum spanning tree based on cgMLST analysis of 241 VREfm isolates. Nodes are colored by ST (number of isolates per ST shown in legend). Isolates with ≤20 allelic differences were grouped into the same cluster; collapsed nodes are shown as circles, with node size proportional to the number of isolates within the cluster. STs represented by a single isolate are shown in white. Branch lengths are proportional to the number of allelic differences.

After excluding six isolates with ≥10% missing alleles, the remaining 241 VREfm isolates were analyzed by cgMLST. A minimum spanning tree was constructed using GrapeTree (MSTree V2 algorithm) (**Figure 2**). Nodes were colored by sequence type (ST), with STs represented by a single isolate shown in white. Three predominant clusters were identified: cluster 1 (ST80, n=41, CT8524), cluster 2 (ST363, n=30), and cluster 3 (ST547, n=19, CT7977). These three clusters formed distinct, well-separated clades in the tree, while the remaining STs were interspersed across the tree, often as singletons or small clusters. Branch lengths are proportional to the number of allelic differences.

## Discussion

*E. faecalis* and *E. faecium* are both important clinical pathogens and in China they were the second and third most frequently reported gram-positive bacteria. According to previous studies, resistance to vancomycin among *E. faecium* was more serious than *E. faecali*s, which is consistent with our findings (247 VREfm vs. 4 VREfs). More than 98% of VREfm in this study belonged to CC17, which is a major cause of hospital-associated *E.faecium* infection (Werner et al., 2020). According to a global genomic study (Yan et al., 2025), which analyzed 1,071 clinical VREfm isolates carrying *van* genes, ST17 (8.1%) was the most common, followed by ST117 (6.1%) and ST78 (5.7%). The distribution of ST types varies across different countries. For instance, ST117 is the most predominant ST in Germany (Xanthopoulou et al., 2020), whereas a high diversity of STs, including ST17, ST80, and ST18, has been observed in the United States (Mills et al., 2025). According to previous studies, the most predominant ST among VREfm isolates in Beijing was ST78, however, in this study ST80 was most common, which was found in most hospitals of Beijing (Sun et al., 2019; Yan et al., 2023; Yang et al., 2015, 2016). This may suggest a potential shift in the predominant ST of VREfm in this region. The shift in ST distribution is not a rare event; rather, it reflects a dynamic and ongoing process of lineage replacement. Analysis of 15,631 global VREfm genomes collected between 2002 and 2022 revealed that ST80 and ST117 have progressively replaced ST17 as the dominant lineages worldwide (Mills et al., 2025). A multicenter study by Shen et al. further supports the emergence of ST80 in China. In Guangdong Province, ST17 was dominant before December 2020 (55%, 11/20), but was replaced by ST80 during 2021-2023, which accounted for 88.63% (195/220) of VREfm isolates, indicating a regional outbreak. In other provinces, ST78 was the most prevalent ST from 2014 to 2021 (32.56%, 14/43), whereas ST80 became the dominant ST in 2022-2023 (32.14%, 9/28). These findings, together with our observation that ST80 has become the most common ST in Beijing, suggest that ST80 is emerging as a dominant VREfm lineage across multiple regions in China (Shen et al., 2024). Fioriti et al. reported a similar changing trend of VREfm isolated from a regional hospital in Italy between 2001 and 2018; the most common STs were ST78 from 2001 to 2007, ST117 in 2016, then ST80 in 2017 and 2018 (Fioriti et al., 2020). The shift may be related to the greater adaptability of ST80 to the hospital environment.

To date, 9 acquired glycopeptide resistance genes (*vanA*, *vanB*, *vanC*, *vanD*, *vanE*, *vanG*, *vanL*, *vanM* and *vanN*) have been reported; *vanA*, *vanB* and *vanM* are more clinically important because they mediate high levels of vancomycin resistance and can be transferred *in vitro* (Onaran Acar et al., 2023). *VanA* followed by *vanB* was the most dominant worldwide. In recent years, a shift from *vanA* genotype to *vanB* genotype has been observed in some countries such as Germany (Werner et al., 2020). *VanM* was first identified in a clinical VREfm isolate collected from Shanghai in 2006, which encoded 343 amino acids with 79.9% identity with VanA protein; *vanM* has been identified in patients from China, Japan, Singapore, and recently in animals from Turkey (Chen et al., 2015; Onaran Acar et al., 2023; Xu et al., 2010).

In China, the prevalence of *van* genes among VRE isolates varied in different cities. *VanA* was the most dominant among VRE isolates in most cities in China, such as Beijing, Nanjing, Chengdu, while *vanM* was more prevalent than *vanA* in Shanghai since 2011 (Chen et al., 2015; Sun et al., 2019; Yan et al., 2023; Yang et al., 2016, 2019; Zhou et al., 2020). In this study, more than 70% of VRE isolates harbored the *vanA* gene; 0.8% (2/247) and 26.3% (65/247) of VREfm carried *vanM*, both *vanA* and *vanM* genes, respectively. Consistent with previous studies, *vanA* was still the most prevalent *van* type in Beijing. Interestingly, *vanA* coexistence with *vanM* was increased compared to a previous study, which reported that 11.9% of VREfm isolates collected from a tertiary teaching hospital in Beijing from 2011 to 2017 harbored the two genes (Sun et al., 2019). Recently, Shen et al. reported that 7.51% of VREfm isolates from a multicenter study involving 19 hospitals across 6 provinces in China co-harbored *vanA* and *vanM* genes (Shen et al., 2024). To date, VRE carrying both *vanA* and *vanM* were only found in China. However, the co-emergence of *vanA* and *vanB* has been reported in a number of studies. VRE isolates carrying two *van* genes were first reported in England, then in Finland, Australia, Saudi Arabia, France, Greece and Poland (Al-Ahdal et al., 2012; Dendle et al., 2009; Marcadé et al., 2014; Papagiannitsis et al., 2017; Suppola et al., 1999; Wardal et al., 2023; Woodford et al., 1997). In most cases, these isolates were associated with an outbreak of VRE (Dendle et al., 2009; Marcadé et al., 2014; Suppola et al., 1999; Woodford et al., 1997). In this study, *vanA*^+^/*vanM*^+^ isolates were predominant in hospital E and 80.5% (29/36) were ST80. Among the 41 ST80 isolates in cluster 1 (**Figure 2**), 26 (63.4%) were collected from hospital E, suggesting a potential outbreak in this hospital. However, a limitation of this study is that the genetic context of *vanA* and *vanM* (plasmid vs. chromosome) could not be determined using short-read WGS data alone, leaving the potential for horizontal gene transfer unclear. Further investigation of vanA+/vanM+ isolates is warranted.

For VRE isolates, two major phenotypes including VanA and VanB have been described. The VanA phenotype, encoded by the *vanA* gene clusters, is characterized by high-level resistance to both vancomycin and teicoplanin, while the VanB phenotype confers moderate to high resistance to vancomycin but susceptibility to teicoplanin (Arthur and Courvalin, 1993). VRE isolates carrying *vanA* gene but susceptible to teicoplanin have been identified in China, Japan, Korea and Egypt, and the phenomenon was termed as *vanA* genotype-vanB phenotype (Gu et al., 2009; Hashimoto et al., 2000; Khairy et al., 2019; Shen et al., 2016; Song et al., 2006). In this study, 89.1% of VREfm and all VREfs isolates were resistant to both vancomycin and teicoplanin; 10.9% VREfm revealed *vanA* genotype-vanB phenotype. A number of studies have investigated the mechanism underlying the discrepancy between genotype and phenotype, however, it remains unclear. The phenomenon may be attributed to genetic variation in *vanA* gene clusters or decreased expression level of *vanA*.

## Conclusion

Although we did not distinguish between infection and colonization, our findings remain clinically relevant for infection control and antimicrobial resistance surveillance, as both infected and colonized patients contribute to VREfm transmission in healthcare settings. The prevalence of VRE, especially VREfm, was more serious in Beijing than other cities in China. ST80 was the most dominant in this study, which is different from previous studies. The increased trend of concomitance of *vanA* and *vanM* was noteworthy.

## Data Availability

The original contributions presented in the study are included in the article/Supplementary Material. Further inquiries can be directed to the corresponding author.
